# Characteristics of the *Mycoplasma pneumoniae* Epidemic from 2019 to 2020 in Korea: Macrolide Resistance and Co-Infection Trends

**DOI:** 10.3390/antibiotics12111623

**Published:** 2023-11-13

**Authors:** Soyoun Shin, Sunhoe Koo, Yong-Jin Yang, Ho-Jae Lim

**Affiliations:** 1 Daejeon & Chungcheong Reference Lab., Seegene Medical Foundation, Daejeon 35203, Republic of Korea; shkoo3388@mf.seegene.com; 2 Department of Molecular Diagnostics, Seegene Medical Foundation, Seoul 04805, Republic of Korea; yjyang@mf.seegene.com (Y.-J.Y.); 52rotc.hjl@mf.seegene.com (H.-J.L.)

**Keywords:** *Mycoplasma pneumoniae*, co-infection, macrolide resistance, SARS-CoV-2, epidemic, community-acquired pneumonia

## Abstract

*Mycoplasma pneumoniae*, a major etiological agent of community-acquired pneumonia, exhibits distinct cyclic epidemic patterns recurring every three to five years. Several cases of co-infection with severe acute respiratory syndrome coronavirus 2 have been reported globally, resulting in unfavorable clinical manifestations. This study investigated the epidemiological features of the recent *M. pneumoniae* outbreak (May 2019–April 2020) using retrospective data from the last five years. Molecular test data for macrolide resistance and co-infection were obtained from the Seegene Medical Foundation. National medical expenditure and hospitalization rates were analyzed using data from The Health Insurance Review and Assessment Service of Korea. The macrolide resistance rate was 69.67%, peaking at 71.30% during the epidemic period, which was considerably higher than the 60.89% rate during non-epidemic periods. The co-infection rate with other respiratory pathogens was 88.49%; macrolide-resistant *M. pneumoniae* strains showed a 2.33% higher co-infection rate than the susceptible strains. The epidemic period had 15.43% higher hospitalization and 78.27% higher medical budget expenditure per patient than non-epidemic periods. The increased rates of macrolide resistance and co-infection observed in macrolide-resistant *M. pneumoniae* during the epidemic period highlight the importance of monitoring future outbreaks, especially considering macrolide resistance and the risk of co-infection with other pathogens.

## 1. Introduction

*Mycoplasma pneumoniae* is a leading cause of bacterial community-acquired pneumonia (CAP) in children and young adults, accounting for approximately 40% of all CAP cases in this age group, with up to 18% of these individuals requiring hospitalization. Macrolides are the first-line therapy and are widely used for *M. pneumoniae* pneumonia (MPP). Since the first reports of widespread macrolide-resistant *M. pneumoniae* (MRMP) appeared in Japan in the early 2000s, subsequently spreading through Asia and eventually to Europe and North America, macrolide-resistance has significantly increased worldwide, resulting in refractory cases with severe and complicated clinical features that necessitate immunomodulating therapy. Asia, including China, Japan, and Korea, has shown a much higher macrolide resistance rate, ranging from 60 to 90% and over 90% to 100% in some regions or periods compared with Europe, where prevalence is substantially lower at 1% to 30% varying from country to country [1,2,3,4,5,6,7,8,9,10,11,12,13,14,15,16,17].

Point mutations at nucleotide positions 2063 and 2064 of the peptidyl-transferase loop of domain V of 23S rRNA have emerged and are associated with high macrolide resistance in *M. pneumoniae* [6]. PCR-based molecular tests for *M. pneumoniae* are particularly specific and sensitive, allowing simultaneous detection of multiple targets and mutations related to antibiotic resistance, especially when processing large numbers of samples [11,18]. However, in Korea, molecular diagnostic kits for macrolide resistance testing for *M. pneumoniae* are not commercially available, and they are not covered by Korea’s national health insurance system yet, thus making it impossible to implement an organized surveillance program like other countries [19]. 

MPP exhibits an endemic transmission pattern characterized by cyclic epidemics occurring every three to five years. Significant MPP outbreaks were reported in Korea from 2006 to 2007, in 2011, and from 2015 to 2016 [13,19]. Although these studies were based on data from a single institute, they consistently reported a rapid increase in macrolide resistance rates of *M. pneumoniae* in Korea, indicating the urgent need for expanded nationwide surveillance to assess the characteristics of macrolide resistance in MPP cases. Since the previous outbreak in the period of 2015–2016, the Seegene Medical Foundation has been providing macrolide-resistance molecular testing for *M. pneumoniae*-positive cases detected with respiratory multiplex panel tests, considering the clinical significance of macrolide resistance in MPP. 

Significant rates of co-infection of *M. pneumoniae* with other bacteria and viruses have been reported, and in some cases, these co-infections result in severe illnesses. Several studies have documented cases of severe acute respiratory syndrome coronavirus 2 (SARS-CoV-2) co-infection with *M. pneumoniae*; the affected patients exhibit severe clinical features and have unfavorable outcomes. Current studies on co-infection are limited and do not adequately consider the cyclic epidemic features of *M. pneumoniae*, resulting in an underestimation of the severity of concurrent outbreaks. Given the ongoing impact of the COVID-19 pandemic, it is crucial to pre-emptively monitor future epidemics involving MPP as a potential co-infection pathogen, especially in young age groups. This requires a comprehensive understanding of the characteristics of the recent outbreaks [20,21,22,23,24,25,26,27,28,29,30,31,32].

This study aimed to characterize the most recent epidemic of MPP in Korea that occurred between 2019 and 2020, which has not been previously investigated. Features of macrolide resistance and co-infection with other pathogens were analyzed utilizing a data set comprising laboratory test data accumulated from May 2017 to April 2022, which were obtained from the Seegene Medical Foundation, Korea, a representative commercial laboratory with a nationwide test requesting system. Additionally, we analyzed medical expenditures and hospitalization rates using the open data system of the Health Insurance Review and Assessment Service system (HIRA, Wonju, Republic of Korea) covering medical services for all Korean citizens, for a comprehensive nationwide study [33].

## 2. Results

### 2.1. Respiratory Panel Test Results Accumulated over a Five-Year Period, Including the Recent Outbreak of M. pneumoniae

**Figure 1:**
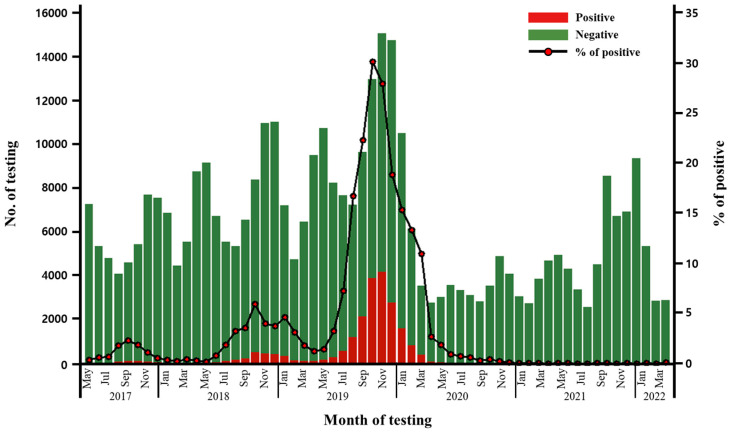
*M. pneumoniae*-positive case numbers among respiratory panel tests performed from May 2017 to April 2022.

The total number of respiratory bacterial panel tests requested during the five years from May 2017 to April 2022 was 376,946. The number of requested tests exhibited a bimodal peak pattern annually, with increases observed between April and May and November and December, reflecting the seasonal prevalence of acute respiratory infections. This trend continued until early 2020, prior to the onset of the SARS-CoV-2 crisis in Korea when the SARS-CoV-2 test was prioritized (Figure 1).

For bacterial panel tests, *S. pneumoniae* (216,433 cases, 57.42%) and *H. influenzae* (128,448 cases, 34.08%) were the most frequently detected. *M. pneumoniae* was identified in 21,331 cases (5.66%). This was followed by *C. pneumoniae* (1.483 cases, 0.39%), *B. pertussis*/*parapertussis* (511 cases, 0.14%), and *L. pneumophila* (185 cases, 0.05%; Table 1).

Notably, *M. pneumoniae*-positive cases demonstrated a sharp peak from May 2019 to April 2020, representing the recent epidemic outbreak. A total of 17,950 cases were identified during the defined epidemic outbreak, accounting for 84.15% of all 21,331 cases detected during the five-year period in the laboratory database. Based on monthly data, the peak was at 4169 cases in November 2019, which accounted for 27.80% of 14,994 cases, of all respiratory bacterial panel tests performed during that month (Figure 1). 

Of the 376,946 bacterial panel tests conducted, 270,261 underwent parallel respiratory virus panel testing. Of these, 247,341 (91.52%) tested positive for at least one of the 16 viruses that were screened. Human rhinovirus was the most frequently detected (37.06%), followed by respiratory syncytial virus A/B (17.74%), adenovirus (15.23%), parainfluenza 1/2/3/4 (14.51%), human bocavirus (13.41%), influenza A/B (6.26%), human enterovirus (6.19%), non-SARS-CoV-2 coronaviruses (4.98%), and human metapneumovirus (4.45%; Table 1). 

### 2.2. Comparison of Demographic and Macrolide Resistance Characteristics of M. pneumoniae-Positive Cases between MPP Epidemic and Non-Epidemic Periods

The average *M. pneumoniae* identification rates were 51.32% for males and 48.68% for females. Young adolescents and children were predominantly affected by *M. pneumoniae* infection. Individuals aged under 15 years accounted for 93.18% of the *M. pneumoniae*-positive cases, and those under the age of 10 years accounted for 85.88%, according to the five-year laboratory data. Similarly, during the epidemic period from May 2019 to April 2020, the prevalence was 93.59% and 86.17% for those under the age of 15 and 10 years, respectively. 

Among the overall 21,331 *M. pneumoniae*-positive cases, 21,222 cases were tested for macrolide resistance and subjected to further analysis. The overall macrolide resistance rate was 69.67% over the five-year study period. The resistance rate peaked at 71.30% during the *M. pneumoniae* epidemic period, which was substantially higher than that observed during non-epidemic periods (60.89%), ranging from 25.0 to 63.10%. The A2063G mutation was detected in 99.47% of macrolide-resistant cases, either as a sole mutation or combined with the A2064G mutation. The A2064G mutation was rarely detected, occurring in only 0.56% of cases, either in isolation or in combination with A2063G (Table 2).

### 2.3. Comparison of M. pneumoniae Co-Infections between the MPP Epidemic and Non-Epidemic Periods

Overall, a high rate of co-infection (18,780 cases, 88.49%) with other pathogens was observed. Among these cases, the most frequently detected bacterial pathogen was S. pneumoniae (10,527 cases, 49.60%) followed by *H. influenza* (9453 cases, 44.54%). Both are well-known strains that are part of normal flora; therefore, clinical correlation is required for the interpretation of its clinical significance. As non-normal floral pathogenic strains, 183 total cases including 158 cases of C. pneumoniae, 23 cases of B. pertussis/parapertusis, and 2 cases of L. pneumophila were co-identified in 0.86% of overall *M. pneumoniae* positive cases. Overall, 84.7% (155 of 183 cases) of these co-infection cases were identified during the epidemic period. No case was observed in which two or more than two non-normal floral pathogenic bacterial strains were co-identified in *M. pneumoniae*-positive cases. For paralleled viral panel tests, 39.31% (8343 of 21,222 cases) of cases had a high rate of co-infection with various virus strains and 14.27% (3208 of 21,222 cases) of cases had a multi-co-infection with two or more than two virus strains. Human rhinovirus was the most frequently detected at 27.28%. In addition to human rhinovirus, respiratory syncytial virus A/B, adenovirus, parainfluenza 1/2/3/4, and human bocavirus showed relatively lower detection rates as co-infection at 5.14%, 6.35%, 3.86%, and 3.82% (Table 3), compared with the overall detection rate identified as 37.06%, 17.74%, 15.23%, 14.51%, and 13.41% in respiratory virus panel tests during the five years, as previously shown in Table 1. The epidemic and non-epidemic periods did not show differences in co-infection rates with various virus strains. 

### 2.4. Co-Infection Characteristics of Macrolide-Resistant and Macrolide-Susceptible M. pneumoniae

We compared co-infection rates between macrolide-resistant *M. pneumoniae* (MRMP) and macrolide-susceptible *M. pneumoniae* (MSMP). MRMP showed 2.33% higher co-infection rates with most other pathogens consistently, except influenza and HMPV, than MSMP (Table 4).

### 2.5. Korean National Health Data on MPP from May 2017 to April 2022

This study was conducted using data from the Health and Medical Big Data Open System of HIRA. The Korean national health insurance (NHI) system was first introduced in Korea in 1977, and it was expanded to cover all citizens in 1989. HIRA sets the scope and standards of services covered by the NHI and allows open access to all the data through a big data platform [34,35]. The national data on MPP cases reported during the same period, from May 2017 to April 2022, were extracted from the HIRA database and analyzed. In total, 224,830 MPP cases were diagnosed and treated during the five-year period nationwide. The reported incidence in females was 5.58% higher than that in males (Table S1). 

The HIRA data consistently defined the epidemic outbreak of the MPP with a sharp increase during the same epidemic period identified based on the laboratory data, with the highest number of cases recorded in November 2019. A total of 88,066 cases were recorded during the defined epidemic outbreak, accounting for 39.17% of all 224,830 cases reported during the five-year period in the HIRA database. Based on monthly data, the number of MPP cases recorded in 2019 doubled from 3772 cases in June to 8136 in September; this figure doubled again to 16,260 in November. The overall average hospitalization rate during the five-year period was 34.88%. During the epidemic period, the hospitalization rate reached its highest point at 44.27%, significantly exceeding the annual average hospitalization rates of the non-epidemic period of 28.84%, which ranged from 14.73 to 33.25% (*p* < 0.001).

The total medical budget reimbursed by the Korean national health insurance program during the five-year period was KRW 91,880 million (approximately USD 70 million), of which 94% was due to hospitalization costs. Among the medical expenses related to MPP, 53.45% were incurred during the epidemic period from May 2019 to April 2020, which can be attributed to the increased number of MPP cases and the higher hospitalization rate of 44.27%. The average medical budget per patient for the entire period was KRW409,000. However, during the epidemic period, the average medical budget per patient increased to KRW 557,677, which was 78% higher than the average for the non-epidemic period (KRW 312,715) and 36.43% higher than the average for the entire five-year period (*p* < 0.001) (Table 5).

## 3. Discussion

This study aimed to examine trends in the prevalence of macrolide resistance and co-infection of *M. pneumoniae* with other pathogens in the May 2019 to April 2020 Korean epidemic, which has not been previously investigated. We found that *M. pneumoniae* exhibits a high macrolide-resistance rate of 69.67% and an overall high co-infection rate of 88.49% with other pathogens. Importantly, the epidemic period showed a higher macrolide-resistance rate, and macrolide-resistant *M. pneumoniae* showed a higher co-infection rate with other pathogens, which were presumably related to the higher hospitalization rate and medical budget expenditure during the epidemic period observed in the national HIRA data.

First, our data highlight *M. pneumoniae* as an important etiological agent of respiratory disease in Korea over the last five years, defining the recent outbreak of *M. pneumoniae* from May 2019 to April 2020 right before the SARS-CoV-2 crisis. A combined analysis of the accumulated laboratory data and national data demonstrated the characteristics of the recent epidemic outbreak of MPP, allowing a comprehensive understanding of the epidemiological characteristics of nationwide scope compared with the previous reports of single institute-based studies [13,19]. Both data sets showed a consistently increased prevalence of MPP during the epidemic period. *M. pneumoniae*-positive cases detected during the epidemic period accounted for 84.15% (17,950 out of 21,331) of all cases detected during the overall five-year period in the laboratory molecular data.

In the national HIRA data, the epidemic period accounted for 39.17% or 88,066 out of the total 224,830 cases during the overall period. The variation between 84.15% and 39.17% in each data set can be explained by the reliance of laboratory data on only respiratory multiplexing PCR panel tests, while the HIRA data are based on final reports with clinical diagnosis of MPP based on broad clinical evidence, which could include single-target PCR, serologic tests, or radiological findings with clinical correlation without molecular tests [11]. Additionally, the Seegene Medical Foundation is a commercial laboratory; therefore, its testing scale could be affected by market sharing and governmental medical insurance policies. The overall *M. pneumoniae* cases detected in this laboratory accounted for 10% and 20% of total cases reported in the HIRA database, respectively, for the five years analyzed and the one-year epidemic period.

Second, *M. pneumoniae* exhibited a high macrolide-resistance rate of 69.67% during the last five years. It was notable that the macrolide-resistance rate during the epidemic period peaked at 71.30%, considerably higher than the 60.89% (range: 25.0–63.10%) of non-epidemic periods. The transition mutation A2063G was most common, concordantly with previous reports. Mutation of the 23S rRNA gene can change macrolide susceptibility; a single mutational event can result in highly resistant strains because *M. pneumoniae* only has a single copy of the 23S rRNA gene [11,36]. Globally, macrolide resistance in *M. pneumoniae* has been increasing for over two decades and is reported to be as high as 90% in some areas in Japan and China and 30% in areas in Europe, emphasizing the necessity of organized surveillance and antimicrobial stewardship. The macrolide-resistance rate of 69.67% found in the recent epidemic in Korea is much higher than the resistant rate in Western countries, which was substantially lower at 1% to 30%, varying from country to country, but similar to the rates ranging from 60 to 90% in Japan and China, which are geographically nearer [1,2,3,4,5,6,7,8,9,10,11,12,13,14,15,16,17]. Because macrolide resistance testing for *M. pneumoniae* is not covered by Korea’s NHI system and molecular diagnostic kits are not commercially available in Korea, the assessment of nationwide macrolide resistance in *M. pneumoniae*-positive cases has been inaccessible. Previous epidemic studies have primarily relied on data from a single clinical institute. These studies have consistently reported a rapid increase in macrolide resistance rates in *M. pneumoniae* of 14.7% in 2006, 51.6% in 2011, and 84.6% in 2015 during consecutive epidemics in Korea. These findings highlight the urgent need for nationwide surveillance to assess macrolide resistance in MPP cases [13,19]. Considering the clinical significance of macrolide resistance during the epidemic outbreak in 2015, the Seegene Medical Foundation developed an in-house method for macrolide-resistance testing and provided additional testing for all *M. pneumoniae*-positive cases in requested respiratory panel tests. These results are based on an analysis of five years’ worth of accumulated laboratory data from a nationwide test request service system, making them valuable and comprehensive; it is not possible to use clinical outcomes in the HIRA database, which has limited integration with medical records.

Chen et al. reported that patients infected with MRMP had a longer febrile period, length of hospital stay, antibiotic drug courses, and defervescence time after macrolide treatment compared with patients infected with MSMP. The risk of fever lasting for >48 h after macrolide treatment was also significantly increased, and an increased proportion of patients was changed to second-line treatment in their meta-analysis, with the final 24 records selected in the qualitative synthesis. Their study emphasized promoting antibiotic stewardship to reduce macrolide resistance caused by the selective pressure of a vicious cycle between the extent of *M. pneumoniae* and consequent increased consumption of antibiotic drugs [15]. 

In epidemiologic features, significant correlations between genetic subtypes of P1 and P2 or MLVA type 4-5-7-2 and macrolide resistance, higher expression of virulence factors, and more severe disease have been reported. These highlight that clonal subtype analysis needs to be considered in further study to identify clonal spread and its effect on macrolide resistance and clinical sequences [11,37,38,39,40,41,42,43]. 

Third, overall, a high rate of co-infection (88.49%) with other pathogens was observed. Notably, MRMP showed higher co-infection rates than MSMP, regardless of epidemic or non-epidemic periods, consistent with the trends in most viruses. Among these cases, the most frequently detected bacterial pathogen was *S. pneumoniae* (10,527 cases, 49.60%) followed by *H. influenza* (9453 cases, 44.54%). Both are well-known strains that are part of the normal flora; therefore, clinical correlation is required for the interpretation of their clinical significance. A total of 0.86% of *M. pneumoniae*-positive cases showed co-infection with non-normal floral pathogenic strains including 158 cases of *C. pneumoniae*, 23 cases of *B. pertussis/parapertusis*, and 2 cases of *L. pneumophila*, and most co-infection cases were identified during the epidemic period. *M. pneumoniae*-positive cases showed a high co-infection rate of 39.31% with various virus strains and multi-co-infection of 14.27% with two or more virus strains. Among the parallel virus panel tests, HRV was most commonly co-identified at 27.28%, followed by adenovirus, RSV, and parainfluenza. These findings are concordant with the general detection rate frequency in the virus panel test performed. Generally, co-infection with viral strains may not affect the primary antibacterial treatment regimen and also for *C. pneumoniae*, the most common pathogenic bacterial pathogenic strain, the treatment of choice is not different from *M. pneumoniae* infection as atypical pneumonia. Macrolide resistance in *C. pneumoniae* infections has been rarely reported [44,45]. The clinical effects and outcomes of various combinations of co-infection or multi-co-infection with different bacterial and viral strains need to be considered in further studies of prospective clinical cohort design and national surveillance systems. 

Given the ongoing impact of the SARS-CoV-2 pandemic, it is crucial to closely monitor co-infection trends with other respiratory pathogens. Several studies have documented instances of SARS-CoV-2 co-infection with other respiratory pathogens including *M. pneumoniae*, presenting severe clinical features and unfavorable outcomes. It is worth noting that some studies may not have adequately considered the cyclic epidemic patterns of *M. pneumoniae* in their analyses [20,21,22,23,24,25,26,27,28,29,30,31,32]. Most symptomatic patients with SARS-CoV-2 infection develop atypical pneumonia, characterized by fever, cough, and shortness of breath, which poses a challenge in making differential diagnoses based solely on clinical presentation. Indeed, co-infections with *M. pneumoniae* are likely to go unnoticed without active evaluation [46,47]. Considering the clinical context, *M. pneumoniae* is one of the most important potential co-infection pathogens. 

Consistent with previous reports, MPP was found to be prevalent in children, particularly those under 15 years of age. Notably, this age group has a surprisingly high co-infection rate of SARS-CoV-2 with other pathogens. In fact, out of the total reported infections, 16 out of 34 cases (47.0%) involved co-infection with *M. pneumoniae*. Several case reports have documented *M. pneumoniae* and SARS-CoV-2 co-infections with unfavorable clinical features; for example, a 12-year-old boy presented with SARS-CoV-2 infection with pleural effusion, further complicated by secondary *M. pneumoniae* infection [26,29,30]. These observations highlight the fragility of this age group with the limited availability of SARS-CoV-2 vaccinations [48,49]. This age group exhibited not only susceptibility to *M. pneumoniae* infection but also a high rate of macrolide resistance in *M. pneumoniae* infections. Second-line therapies for refractory MPP include alternative classes of antibiotics, such as doxycyclines, tetracyclines, or fluoroquinolones; however, these drugs are commonly associated with adverse reactions, including gastrointestinal disturbances, esophagitis, photosensitivity, and tooth discoloration, which often preclude their indication for children. In addition, fluoroquinolones are not recommended for children due to concerns related to musculoskeletal adverse events, including arthralgia, arthritis, tendinopathy, and gait abnormality. This class of drugs is not generally approved for children under the age of 12 and is not approved for subjects under the age of 18 in Korea [17,19,46,50,51].

We compared co-infection rates between macrolide-resistant *M. pneumoniae* (MRMP) and macrolide-susceptible *M. pneumoniae* (MSMP). MRMP showed 2.33% higher co-infection rates with most other pathogens consistently, except influenza and HMPV, than MSMP, regardless of epidemic or non-epidemic periods. Further studies for co-infection susceptibility of macrolide-resistant *M. pneumoniae* also need to be considered.

Fourth, HIRA data demonstrated that the epidemic period showed a higher hospitalization rate (44.27% vs. 28.84%) and 78.33% higher medical budget expenditure per patient compared with non-epidemic periods; this could be explained by the higher macrolide-resistance rate during the epidemic period and the higher co-infection rate of MRMP with other viruses. Previous studies showed an association between MRMP and increased use of steroid therapy, more severe or prolonged disease, and prolonged hospitalization periods [13,19,52,53,54,55,56,57,58,59,60].

These findings emphasize the importance of conducting macrolide-resistance testing for *M. pneumoniae* infections and implementing further surveillance efforts that consider co-infection features. These measures are crucial for gaining a more in-depth understanding of the epidemiological factors contributing to higher MRMP rates during epidemic outbreaks and higher co-infection rates with other viruses. Further investigation into the cyclic epidemic features related to macrolide resistance should focus on the following areas: (i) understanding the distinct characteristics of the shift between genetic P1 and P2 subtypes from an immunological perspective; (ii) examining the significantly high resistance levels and severe clinical features of subtypes such as MLVA type 4-5-7-2; (iii) studying the transmission of genetically acquired macrolide resistance; and (iv) investigating the role of virulence factors, including the recently discovered community-acquired respiratory distress syndrome (CARDS) toxin [11,37,38,39,40,41,42,43]. By exploring these aspects, we can gain a better understanding of macrolide resistance patterns and their relation to cyclic epidemics, which can inform the development of effective prevention and treatment strategies. 

Moreover, compared with those in most other countries, Korea’s national data showed high hospitalization rates, which warrants further evaluation compared with MRMP and co-infection features of epidemics in different countries [1,2,3,4,5,6,7,8,9,10,11,12,13,14,15,16,17]. 

The limitation of this study was the inability to directly analyze clinical information due to the inherent characteristics of independent big data sets, which are separated from clinical records as a result of personal information protection regulations in Korea. However, this study provides comprehensive and reliable prevalence data for 224,830 MPP cases recorded in the national health insurance system. It also offers insights into the laboratory test findings of macrolide resistance and co-infection in 21,222 cases accumulated over five years, which is generally not obtainable.

In conclusion, this study, utilizing data from a nationwide referral laboratory center and the national health insurance database, provided important insights into the recent epidemic outbreak of *M. pneumonia.* The findings revealed high macrolide-resistance rates and co-infection incidence with other pathogens of the recent epidemic on a nationwide scale. Significantly increased macrolide resistance rates in the epidemic period and co-infection rates of MRMP were presumed to be associated with elevated hospitalization rates and significantly increased medical expenditure per patient during the epidemic period observed in the NHI data analysis. The present study emphasizes the importance of epidemiological monitoring to anticipate future cycles of MPP outbreaks that could overlap with the current aftermath of the SARS-CoV-2 pandemic. Rapid molecular assessment for macrolide resistance and evaluations for co-infection trends need to be available in Korea and considered in the primary diagnostic evaluation for adjusting early treatment decisions, preventing future epidemic outbreaks of MPP, and proper antibiotic resistance stewardship. Particular attention should be paid to young children who are disproportionately affected and face limited treatment options for macrolide-resistant MPP infections. 

## 4. Materials and Methods

This study was designed as a collaborative data analysis between the Health Insurance Review and Assessment Service system (HIRA, Korea) and Seegene Medical Foundation (Korea) clinical laboratory testing. This study was approved by the Institutional Review Board of the Seegene Medical Foundation (SMF-IRB-2023-009). Informed consent from the participants was waived because the data collected for this study were anonymized. Data analysis was performed using two nationwide independent big data sets, both of which were collected from May 2017 to April 2022. First, laboratory data on macrolide resistance and co-infection with other pathogens were obtained from the Seegene Medical Foundation, a representative commercial laboratory with a nationwide test requesting system. Second, data on medical expenditures and hospitalization rates of MPP cases were obtained from the open data system of the HIRA, which covers medical services for all Korean citizens [27,28]. Comparisons were made between the characteristics of the laboratory and HIRA data during the epidemic periods and those of the non-epidemic periods preceding and following them. Publicly accessible open-source data on MPP included prevalence rates by age and sex, outpatient treatment and hospitalization rates, and medical expenditures reimbursed by the national health insurance program.

### 4.1. Respiratory Panel Test and Co-Infection Analysis

Seeplex™ PneumoBacter ACE Detection/Allplex™ PneumoBacter (Seegene, Seoul, Korea) assays were used for the detection of respiratory bacteria, and Anyplex™ II RV16 Detection/Allplex™ Respiratory Panel (Seegene) kits were used for the detection of respiratory viruses [61,62,63,64,65]. Six bacteria and 16 respiratory viruses were included: *Bordetella pertussis*, *Chlamydia pneumoniae*, *Haemophilus influenzae*, *Legionella pneumophila*, *M. pneumoniae*, and *Streptococcus pneumoniae* for bacterial panel tests, and adenovirus, human bocavirus, coronavirus OC43/NL63/229E, human enterovirus, human metapneumovirus, influenza A/B, parainfluenza type 1/2/3/4, respiratory syncytial virus A/B, and human rhinovirus for virus panel tests. Since July 2019, *Bordetella parapertussis* has additionally been included in the Allplex™ PneumoBacter assay (Seegene) kit.

A total of 376,946 case data points with respiratory bacterial panel tests completed over the last five years from May 2017, since the macrolide-resistance test was performed for *M. pneumoniae*, were subjected to analysis. Among these, 270,261 samples underwent viral panel tests simultaneously.

### 4.2. Detection and Characterization of Macrolide Resistance in M. pneumoniae

Sequencing testing was conducted on most positive cases of *M. pneumoniae* for macrolide resistance. Among the 21,331 *M. pneumoniae*-positive cases, 21,222 cases were evaluated for macrolide resistance using in-house developed realtime PCR. Macrolide-resistance rates were based on the prevalence of mutations (A2063G and A2064G) in the macrolide-resistance region of the 23S rRNA of *M. pneumoniae*. Oligonucleotides specific to macrolide-resistant *M. pneumoniae* (MRMP) were designed based on an *M. pneumoniae* reference sequence (23S rRNA, GenBank accession number X68422.1). The NCBI-Basic Local Alignment Search Tool (NCBI-BLAST) and multAlin interface were used to optimize specificity for *M. pneumoniae* [66]. The sequences used in the MRMP assay are summarized in Table S2. 

The MRMP assay consisted of the following reagents: 10 μL oligonucleotide mixture, 5 μL 4× PCR enzyme mixture, and 5 μL nucleic acid. The amplification procedure was conducted under the following conditions: 95 °C for 15 min (pre-denaturation), followed by 45 cycles of 95 °C for 10 s (denaturation), and 62 °C for 45 s (annealing). The MRMP assay was designed to detect the A2063 mutation using the FAM (Fluorescein) channel, the A2064 mutation using the VIC (Tetrachlorofluorescein) channel, and *M. pneumoniae* using the Texas red channel. To prevent false-negative results, the human hemoglobin subunit beta was co-detected using Cy5 as an internal control. All molecular tests were performed using the CFX96 system (Bio-Rad Laboratories, Inc., Irvine, CA, USA).

### 4.3. Statistical Analysis

R Studio (ver. 4.1.2; R_Studio Inc., Boston, MA, USA) was used to perform all statistical analyses and generate graphs. The significance between the periods and the percentage of patients (hospitalization vs. non-hospitalization) was evaluated using the chi-square test. Medical budget expenditure (per patient, outpatient, and hospitalization) was compared among periods using one-way analyses of variance (ANOVA) with Tukey HSD post hoc tests. The threshold for statistical significance was set at *p*-value ≤ 0.05.

## Figures and Tables

**Table 1:** Five-year accumulated respiratory bacteria/virus panel tests result from May 2017 to April 2022.

Identified Strains	Total Positives from May 2017 to April 2022 (%)	MP in the Epidemic Period from May 2019 to April 2020 (%)	Non-MP in the Epidemic Period (%)
***No.* of bacteria panels tested**	**376,946**	**108,798**	**268,148**
*S. pneumoniae*	216,433 (57.42)	54,597 (50.18)	161,836 (60.35)
*H. influenzae*	128,448 (34.08)	49,068 (45.10)	79,380 (29.60)
*M. pneumoniae*	21,331 (5.66)	17,950 (16.50)	3381 (1.27)
*C. pneumoniae*	1483 (0.39)	587 (0.54)	896 (0.33)
*B. pertussis*/*parapertusis*	511 (0.14)	157 (0.14)	354 (0.13)
*L. pneumophila*	185 (0.05)	59 (0.05)	126 (0.05)
***No.* of virus panels tested**	**270,261**	**78,780**	**191,481**
CoV OC43/229E/NL63	13,451 (4.98)	5060 (6.42)	8039 (4.20)
ADV	41,152 (15.23)	14,464 (18.36)	26,342 (13.76)
RSV A/B	47,951 (17.74)	11,866 (15.06)	36,026 (18.81)
PIV type 1/2/3/4	39,220 (14.51)	11,359 (14.42)	27,657 (14.44)
Inf A/B	16,906 (6.26)	5736 (7.28)	11,170 (5.83)
HMPV	12,030 (4.45)	4727 (6.00)	7298 (3.81)
BoV	36,245 (13.41)	9315 (11.82)	26,614 (13.90)
HEV	16,727 (6.19)	7900 (10.03)	8818 (4.61)
HRV	100,158 (37.06)	30,459 (38.66)	67,938 (35.48)

Abbreviations: MP, *M. pneumoniae*; CoV, coronaviridea; ADV, adenovirus; RSV, human respiratory syncytial virus; PIV, parainfluenza; Inf, influenza; HMPV, human metapneumovirus; BoV, human bocavirus; HEV, human enterovirus; HRV, human rhinovirus.

**Table 2:** Macrolide resistance-associated mutations in *M. pneumoniae*.

Period	MRMP/MP-Positive (%)	A2063G Sole	A2064G Sole	Combined Mutation
May 2017–April 2018	290/561 (51.69)	285	5	0
May 2018–April 2019	1621/2579 (62.85)	1600	21	0
May 2019–April 2020	12,767/17,906 (71.30)	12,712	51	4
May 2020–April 2021	106/168 (63.10)	104	2	0
May 2021–April 2022	2/8 (25.00)	2	0	0
Total	14,786/21,222 (69.67)	14,703	79	4

Abbreviations: MRMP, macrolide-resistant *M. pneumoniae*.

**Table 3:** Co-infection characteristics of *M. pneumoniae* with other pathogens.

Identified Strains	Total Positives from May 2017 to April 2022 (%)	MP in the Epidemic Period from May 2019 to April 2020 (%)	Non-MP in the Epidemic Period (%)
***No. of M. pneumoniae* single infection**	2442 (11.51)	2179 (12.17)	263 (7.93)
***No. of M. pneumoniae* co-infection**	18,780 (88.49)	15,727 (87.83)	3053 (92.07)
*S. pneumoniae*	10,527 (49.60)	8200 (45.79)	2327 (70.17)
*H. influenzae*	9453 (44.54)	8348 (46.62)	1105 (33.32)
*C. pneumoniae*	158 (0.74)	136 (0.76)	22 (0.66)
*B. pertussis*/*parapertusis*	23 (0.11)	17 (0.09)	6 (0.18)
*L. pneumophila*	2 (0.01)	2 (0.01)	0 (0)
CoV OC43/229E/NL63	834 (3.93)	704 (3.93)	130 (3.92)
ADV	1347 (6.35)	1103 (6.16)	244 (7.36)
RSV A/B	1091 (5.14)	916 (5.12)	175 (5.28)
PIV type 1/2/3/4	819 (3.86)	728 (4.07)	91 (2.74)
Inf A/B	529 (2.49)	467 (2.61)	62 (1.87)
HMPV	101 (0.48)	87 (0.49)	14 (0.42)
BoV	811 (3.82)	616 (3.44)	195 (5.88)
HEV	1085 (5.11)	940 (5.25)	145 (4.37)
HRV	5789 (27.28)	4967 (27.74)	822 (24.79)

Abbreviations: MP, *M. pneumoniae*; CoV, coronaviridea; ADV, adenovirus; RSV, human respiratory syncytial virus; PIV, parainfluenza; Inf, influenza; HMPV, human metapneumovirus; BoV, human bocavirus; HEV, human enterovirus; HRV, human rhinovirus.

**Table 4:** Co-infection characteristics of macrolide-resistant and macrolide-susceptible *M. pneumoniae* with other pathogens.

Identified Strains	MRMP (%)	MSMP (%)
*M. pneumoniae* single infection	1597 (10.80)	845 (13.13)
*M. pneumoniae* co-infection	13,189 (89.20)	5591 (86.87)
*S. pneumoniae*	7448 (50.37)	3079 (47.84)
*H. influenzae*	6651 (44.98)	2802 (43.54)
*C. pneumoniae*	116 (0.78)	42 (0.65)
*B. pertussis*/*parapertusis*	17 (0.11)	6 (0.09)
*L. pneumophila*	1 (0.01)	1 (0.02)
CoV OC43/229E/NL63	634 (4.29)	200 (3.11)
ADV	954 (6.45)	393 (6.11)
RSV A/B	802 (5.42)	289 (4.49)
PIV type 1/2/3/4	603 (4.08)	216 (3.36)
Inf A/B	358 (2.42)	171 (2.66)
HMPV	69 (0.47)	32 (0.50)
BoV	597 (4.04)	214 (3.33)
HEV	845 (5.71)	240 (3.73)
HRV	4212 (28.49)	1577 (24.50)

Abbreviations: MRMP, macrolide-resistant *M. pneumoniae*; MSMP, macrolide-susceptible *M. pneumoniae*; MP, *M. pneumoniae*; CoV, coronaviridea; ADV, adenovirus; RSV, human respiratory syncytial virus; PIV, parainfluenza; Inf, influenza; HMPV, human metapneumovirus; BoV, human bocavirus; HEV, human enterovirus; HRV, human rhinovirus.

**Table 5:** National data on *M. pneumoniae* cases in Korea obtained from the HIRA database from May 2017 to April 2022.

Periods	Patient Numbers Reported			Medical Budget Expenditure Reimbursed by the Korean Government Insurance Program (Thousand Won)			
	Total	Outpatient Cases	Hospitalization Cases	Total	Per Patient	Outpatient Cases	Hospitalization Cases
Total	224,830	165,108	78,431	91,880,621	409	5,509,959	86,370,661
		73.44%	34.88%			6.00%	94.00%
May 2017–Apr 2018	47,451	34,175	15,778	14,815,328	312	1,031,125	13,784,204
	21,11%	72.02%	33.25%	16.12%		6.96%	93.04%
May 2018–Apr 2019	51,533	37,910	16,967	18,701,170	363	1,165,592	17,535,579
	22.92%	73.56%	32.92%	20.35%		6.23%	93.77%
May 2019–Apr 2020	88,066	61,027	38,991	49,112,376	558 *	2,130,684	46,981,691
	39.17%	69.30%	44.27% *	53.45%		4.34%	95.66%
May 2020–Apr 2021	17,056	13,949	3642	5,123,715	300	472,864	4,650,850
	7.59%	81.78%	21.35%	5.58%		9.23%	90.77%
May 2021–Apr 2022	20,724	18,047	3053	4,128,032	199	709,694	3,418,337
	9.22%	87.08%	14.73%	4.49%		17.19%	82.81%
			*p* < 0.001		*p* < 0.001		

Abbreviations: MPP, *Mycoplasma pneumoniae* pneumonia; HIRA, Health Insurance Review and Assessment Service system. * Significant differences between the indicated periods and different periods.

## Data Availability

All data are available within this article.

## Supplementary Materials

The following supporting information can be downloaded at: https://www.mdpi.com/article/10.3390/antibiotics12111623/s1, Supplementary Table S1: Distribution of sex data for HIRA from May 2017 to April 2022; Supplementary Table S2: Oligonucleotide primers and probes for real-time PCR to detect target pathogens and an internal control.
