# Supplementary material for: Characteristics of the Mycoplasma pneumoniae Epidemic from 2019 to 2020 in Korea: Macrolide Resistance and Co-Infection Trends
Source: Antibiotics (Basel). 2023 Nov 13;12(11):1623. doi: 10.3390/antibiotics12111623 (PMC10669541; doi:10.3390/antibiotics12111623)
Supplement: Supplementary file 1 [file antibiotics-12-01623-s001.zip › antibiotics-2683809-supplementary.pdf]

antibiotics

MDPI

Supplementary Results

Supplementary Table S1. Distribution of sex data for HIRA from May 2017 to April 2022

Periods	Total	Male (%)	Female (%)
Total	224,830	106,141 (47.21%)	118,689 (52.79%)
May 2017–April 2018	47,451	21,594 (45.51%)	25,857 (54.49%)
May 2018–April 2019	51.533	23,991 (46.55%)	27,542 (53.45%)
May 2019–April 2020	88,066	42,259 (47.99%)	45,807 (52.01%)
May 2020–April 2021	17,056	8,417 (49.35%)	8,639 (50.65%)
May 2021–April 2022	20,724	9,880 (47.67%)	10,844 (52.33%)

Supplementary Table S2. Oligonucleotide primers and probes for real-time PCR to detect target pathogens and an internal control

Pathogen	Type	Sequences (5′-3′)
M. pneumoniae	F. primer	GCCGTAACTATAACGGTCCTAAG
	R. primer	CCTTTCGCATCAACAAGTCCTA
	23S rRNA probe	Texas red-CGGTGAAATCCAGGTACGGGTGAA-BHQ2
	A2063G probe	FAM-ACGGGAAGACCTCGTGAA-MGBNFQ
	A2064G probe	VIC-CGTGGTCTCTCCGTCC- MGBNFQ
Internal control (HBB)	F. primer	GGCATAAAAGTCAGGGCAGAIIIIICTATTGCT
	R. primer	CCAACTTCATCCACGTTCACCIIIIICCACAGGG
	probe	Cy5-CCTGAGGAGAAGTCTGCCGTTACTGC-BHQ2

Characteristics of Taqman and minor groove binder (MGB) probes with respective quenchers. Taqman probes were labeled with BHQ at the 3′ end (23S rRNA and internal control). Minor groove binder probes were labeled with MGBNFQ at the 3′ end (A2063G and A2064). Abbreviations: F. primer, forward primer; R. primer, reverse primer; probe, fluorescently labeled primer; BHQ, black hole quencher; MGBNFQ, minor groove binder non-fluorescent quencher.
